# Retinal Thinning in Attention Deficit Hyperactivity Disorder (ADHD): Structural Changes Detected by Spectral-Domain OCT

**DOI:** 10.3390/jcm14165723

**Published:** 2025-08-13

**Authors:** Carmen Miquel-Lopez, Jose Javier Garcia-Medina, A. Eusebio Lopez-Hernandez, Diego Garcia-Ayuso, Maravillas De-Paco-Matallana, Javier Hernandez-Olivares, Maria Dolores Pinazo-Duran, Monica Del-Rio-Vellosillo

**Affiliations:** 1 General University Hospital Morales Meseguer, 30008 Murcia, Spain; carmenmiquell@gmail.com (C.M.-L.); mavidepaco@hotmail.com (M.D.-P.-M.); monica.delrio@um.es (M.D.-R.-V.); 2 Department of Ophthalmology, Optometry, Otolaryngology and Pathology, University of Murcia, 30100 Murcia, Spain; antonioeusebio.lopezh@um.es (A.E.L.-H.); diegogarcia@um.es (D.G.-A.); 3 Ophthalmic Research Unit “Santiago Grisolia”, 46010 Valencia, Spain; dolores.pinazo@uv.es; 4 Spanish Net of Inflammatory Diseases RICORS, Institute of Health Carlos III, 28029 Madrid, Spain; 5 Clinical and Experimental Optometry Research Group, Faculty of Optics and Optometry, University of Murcia, 30003 Murcia, Spain; 6 Experimental Ophthalmology Research Group, Department of Ophthalmology, Optometry, Otolaryngology and Pathology, Faculty of Medicine, University of Murcia, Instituto Murciano de Investigación Biosanitaria Pascual Parrilla—IMIB, 30120 Murcia, Spain; 7 University Hospital Virgen de la Arrixaca, 30120 Murcia, Spain; hdezolivares@gmail.com; 8 Cellular and Molecular Ophthalmobiology Group, Surgery Department, Faculty of Medicine and Dentistry, University of Valencia, 46010 Valencia, Spain

**Keywords:** ADHD, optical coherence tomography, retina, macula, optic nerve, thickness, segmentation, RNFL, OCT

## Abstract

**Background/Objectives:** Attention deficit hyperactivity disorder (ADHD) is a highly prevalent neurodevelopmental disorder. As the retina is an extension of the central nervous system, retinal imaging may provide insights into the ADHD pathophysiology. The objective of this work was to evaluate structural retinal alterations using optical coherence tomography (OCT) in ADHD patients compared to neurotypical controls. **Methods**: A case–control study involving 200 eyes (100 from 50 patients with ADHD and 100 from 50 controls) was conducted by comparing the thicknesses of the macular region (total retina, inner and outer retinal layers, ganglion cell layer plus inner plexiform layer [GCIPL], and macular retinal nerve fiber layer [mRNFL]), the peripapillary region (pRNFL), and the optic nerve head (ONH) parameters. Areas under the curve (AUCs) were calculated to evaluate diagnostic performance. Right and left eyes were analyzed separately. **Results**: Patients with ADHD showed a significant reduction in total and outer retinal thickness across several macular sectors in both eyes. No significant differences were observed in mRNFL, GCIPL, inner retina, pRNFL, or ONH parameters between groups. AUC values derived from ROC analysis indicate moderate diagnostic performance for total and outer retinal thickness in the macular region. **Conclusions:** ADHD is associated with retinal thinning in the macula (total and outer retinal thickness) in both eyes, suggesting the potential of OCT-based biomarkers for this condition.

## 1. Introduction

Attention deficit hyperactivity disorder (ADHD) is one of the most prevalent neurodevelopmental conditions, affecting approximately 5–7% of children and adolescents worldwide. Traditionally regarded as a childhood-limited disorder, it is now recognized that a substantial proportion of individuals continue to experience clinically significant symptoms into adulthood, with an estimated adult prevalence of around 2.5%. ADHD is characterized by persistent patterns of inattention, hyperactivity, and impulsivity that interfere with academic, occupational, and social functioning [1].

The etiology of ADHD is multifactorial, with strong genetic contributions and the involvement of multiple brain regions, including the prefrontal cortex, basal ganglia, and cerebellum [2]. Neuroimaging studies have revealed structural and functional alterations in these areas, alongside dysregulation of dopaminergic and noradrenergic neurotransmission [3]. However, despite increasing neurobiological evidence, the diagnosis of ADHD remains primarily clinical, lacking validated biomarkers or objective diagnostic tools.

The retina, as an embryological extension of the central nervous system (CNS), offers a unique window into the brain. It is composed of organized layers of neural cells, including ganglion cells and their axons, which converge to form the optic nerve. Given its shared neurodevelopmental origin with the brain, the retina is being increasingly investigated as a potential biomarker site in neurological and psychiatric conditions. In particular, optical coherence tomography (OCT) is a non-invasive imaging modality that enables in vivo, high-resolution visualization of retinal architecture and is widely used in both research and clinical practice.

Emerging evidence has linked ADHD with structural retinal changes detectable by OCT [4,5,6,7,8,9,10,11,12]. Some studies have reported thinning in specific layers such as the ganglion cell layer and the peripapillary retinal nerve fiber layer (pRNFL), while others have failed to find significant differences. The inconsistency in findings may be due to differences in methodology, sample size, age groups, and OCT protocols. Given the need for objective biomarkers in ADHD and the growing interest in retinal imaging as a neuropsychiatric tool, this study aims to evaluate structural retinal differences between patients with ADHD and neurotypical controls using spectral-domain OCT.

## 2. Materials and Methods

### 2.1. Study Design and Population

A descriptive, prospective, observational case–control study was conducted at the General University Hospital Morales Meseguer (Murcia, Spain) between February 2022 and June 2023. The study included 100 participants aged 10 to 26 years: 50 patients with a confirmed diagnosis of attention deficit hyperactivity disorder (ADHD) and 50 neurotypical controls. ADHD subjects were recruited through the regional ADHD support network “ADAHI” and controls were selected from the hospital healthy patients. All participants provided written informed consent, and the study was approved by the local ethics committee. The research was conducted in accordance with the Declaration of Helsinki and data protection regulations.

### 2.2. Inclusion and Exclusion Criteria

Inclusion criteria for both groups were as follows: Caucasian ethnicity, age between 10 and 26 years, best-corrected visual acuity (BCVA) ≥ 20/40, and refractive error < 6 diopters spherical equivalent and <2.5 diopters of astigmatism. ADHD diagnosis was confirmed by at least two independent specialists according to the same criteria from Diagnostic and Statistical Manual of Mental Disorders, Fifth Edition (DSM-5) [13]. Exclusion criteria included active ocular pathology, congenital ocular malformations, amblyopia, history of ocular disease, poor-quality OCT images (signal strength ≤ 20, motion artifacts, or segmentation errors), or any systemic disease that could affect OCT measurements.

### 2.3. Ophthalmic Examination

All participants underwent a comprehensive ophthalmologic examination, including BCVA (Topcon ACP8—Topcon Corporation, Tokyo, Japan-), autorefractometry performed before and after cycloplegia (Topcon KR 800—Topcon Corporation, Tokyo, Japan), slit-lamp biomicroscopy, dilated fundus examination, and axial length and keratometry measurements using a Pentacam AXL corneal topographer (Oculus Optikgeräte GmbH, Wetzlar, Germany).

### 2.4. OCT Imaging Protocol

Retinal imaging was performed using the Spectral-Domain Optical Coherence Tomography (SD-OCT) system Cirrus 5000 (Carl Zeiss Meditec, Dublin, CA, USA). The Macular Cube 512 × 128 protocol was used to obtain volumetric scans of the macular region (6 × 6 mm), and the Optic Disc Cube 200 × 200 protocol was used for the optic nerve head (ONH) and peripapillary region (also 6 × 6 mm). All scans were performed by the same experienced operator on both eyes of each patient (C.M.L.).

### 2.5. Retinal Structural Analysis

**Figure 1:**
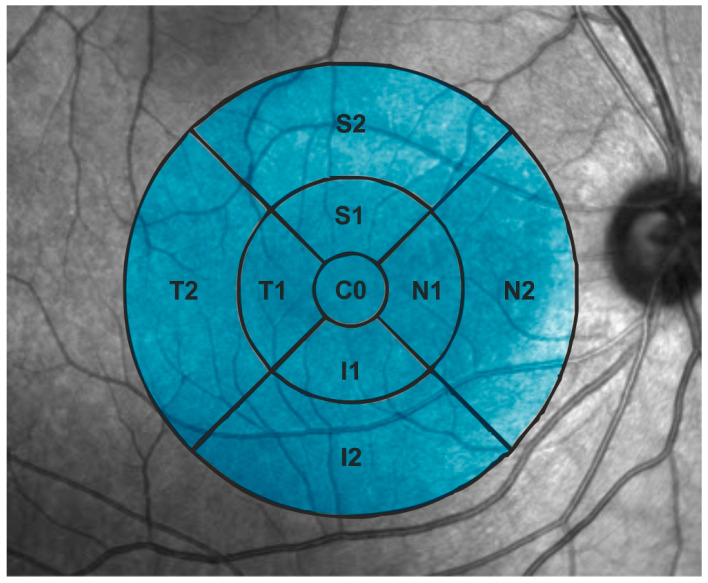
ETDRS grid in a right eye. Note that this grid presents 9 sectors. T = temporal, N = nasal, S = superior, I = Inferior, C0 = fovea. Number 1 and number 2 refer to the inner ring and the outer ring, respectively.

**Figure 2:**
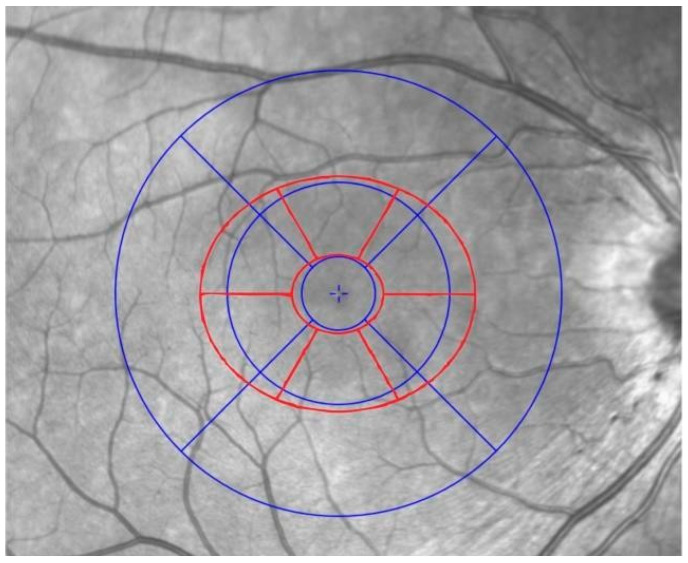
Correspondence between ETDRS sectors (blue) and elliptical sectors (red) in a right eye.

Macular analysis of total retina thickness was conducted using the Early Treatment Diabetic Retinopathy Study (ETDRS) grid, which divides the macular region (6 mm diameter centered on the fovea) into a central subfield and two concentric rings, each further subdivided into four quadrants. This standardized method allows for a detailed and reproducible assessment of retinal thickness across the 9 macular regions (Figure 1 and Figure 2).

In addition, another seven-sector macular elliptical map (Figure 2, depicted in red) was performed considering the following automatic segmentations calculated by the device [14]:

- Macular RNFL (mRNFL).
- Ganglion cell layer plus inner plexiform layer (GCIPL).
- Outer retinal layers (between inner nuclear layer and Bruch’s membrane).

The seven sectors considered were global (G), superior (S), superotemporal (TS), inferotemporal (TI), inferior (I), inferonasal (NI), and superonasal (NS) (Figure 2, in red).

Additionally, we also considered the sum of GCIPL and mRNFL (inner retina) and the sum of inner and outer retina in the elliptical grid for analysis.

**Figure 3:**
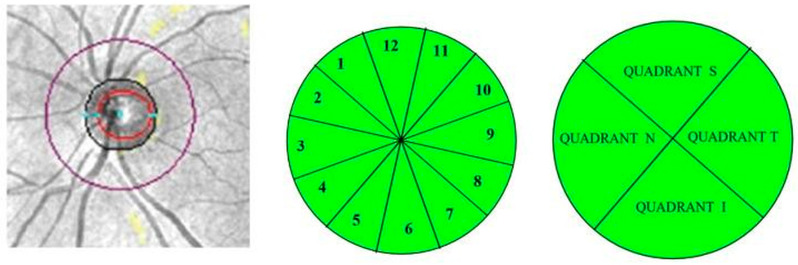
Determination of peripapillary RNFL (left, magenta circle) in 12 clock-hour sectors (middle) and 4 quadrants (right) in a left eye. Superior (S), temporal (T), inferior (I), nasal (N).

Peripapillary RNFL (pRNFL) thickness was also measured in 4 quadrants and in 12 clock-hour sectors around the optic disc (Figure 3).

Finally, we also considered specific optic nerve head parameters obtained by OCT for comparison such as rim area, disc area, average cup-to-disc ratio, vertical cup-to-disc ratio, cup volume, and disc diameter.

### 2.6. Statistical Analysis

Data were exported from the OCT device and analyzed using SPSS (v22.0, SPSS Inc., Chicago, IL, USA) and Python (Version 3.11.4; Python Software Foundation, Wilmington, DE, USA). Right and left eyes were analyzed separately. Data normality was assessed using the Shapiro–Wilk test. Demographic and ophthalmic data comparisons between groups were performed using independent samples Student’s *t*-test. Fisher’s exact test was used to compare sex as a categorical variable. An analysis of covariance (ANCOVA) was performed to compare structural ocular parameters between the case and control groups, using age and sex as covariates. Plus, to account for multiple comparisons across multiple retinal sectors, *p*-values obtained from the ANCOVA were adjusted using the Benjamini–Hochberg false discovery rate (FDR) correction [15]. AUC values from ROC analysis were calculated for structural parameters showing significant between-group differences in both eyes; *p*-values <0.05 were considered statistically significant.

## 3. Results

### 3.1. Demographic and Ophthalmic Data

A total of 112 subjects were initially recruited, of whom 12 were excluded (10 due to poor cooperation, 2 for meeting exclusion criteria). The final sample comprised 100 individuals (200 eyes): 50 ADHD patients (34 males, 16 females) and 50 controls (25 males, 25 females). No statistically significant differences were found between groups in terms of age (22.55 ± 5.56 for control group versus 18.03 ± 6.17 for ADHD group, *p* = 0.058) or sex distribution (*p* = 0.103).

Best-corrected visual acuity (BCVA), spherical equivalent (SE) before and after cycloplegia, axial length (AL), and keratometry parameters (K1, K2) were comparable between groups in both eyes (all *p* > 0.05) (Table 1).

### 3.2. Comparisons of Total Retina Thickness in ETDRS Pattern

**Figure 4:**
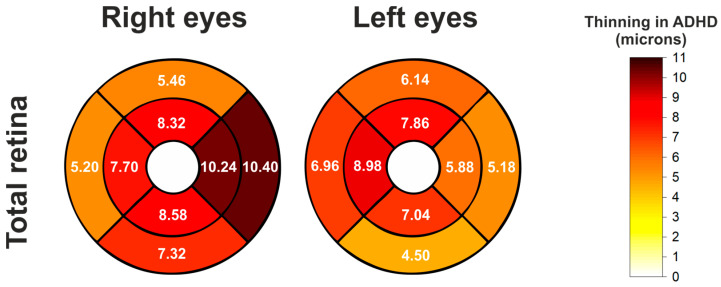
Schematic heatmaps of macular EDTRS grid showing statistically significant thinning (expressed in microns) of the total retina in the ADHD group compared to the control group, for both the right and the left eyes. Mean thickness differences between groups (control group mean thickness minus ADHD group mean thickness) are indicated for each sector where significant differences were found. White sectors indicate no significant differences.

A significant reduction in total retinal thickness was observed in ADHD patients across all ETDRS sectors in both eyes (except the C0 sector). Table 2 contains the comparison data. Figure 4 illustrates these sectoral differences.

### 3.3. AUC for Total Retinal Thickness in the ETDRS Pattern

ROC analysis for total retinal thickness reveals an AUC between 0.6 and 0.7 in most sectors of both eyes, indicating low-to-moderate diagnostic performance (Table 3).

### 3.4. Comparisons of Outer Retinal Layer Thickness in Elliptical Pattern

**Figure 5:**
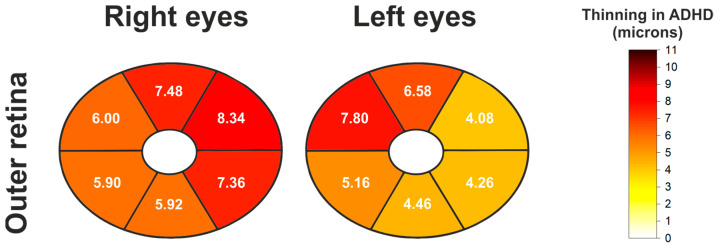
Schematic heatmaps of the macular elliptical grid showing statistically significant thinning (expressed in microns) of the outer retina in the ADHD group compared to the control group, for both right and left eyes. Mean thickness differences between groups (control group mean thickness minus ADHD group mean thickness) are indicated for each sector where significant differences were found. White sectors indicate no significant differences.

To conduct a more detailed analysis, the outer retinal layers (OR) were evaluated separately using the elliptical map pattern. A generalized reduction in outer retinal layer thickness was observed in ADHD patients compared to controls across all sectors. These differences were statistically significant in both the right and left eyes (Table 4 and Figure 5).

### 3.5. AUC for Outer Retina in the Elliptical Pattern

As shown in Table 5, the AUC exceeded 0.7 in most sectors, indicating moderate diagnostic performance of outer retinal thickness in differentiating ADHD patients from controls.

### 3.6. Comparisons of mRNFL, GCIPL, and mRNFL + GCIPL Thickness (Inner Retina) in Elliptical Pattern

No statistically significant differences were detected when comparing mRNFL, GCIPL, and mRNFL + GCIPL thickness (inner retina) between groups.

### 3.7. Comparisons of Inner Retina and Outer Retina in the Elliptical Pattern

**Figure 6:**
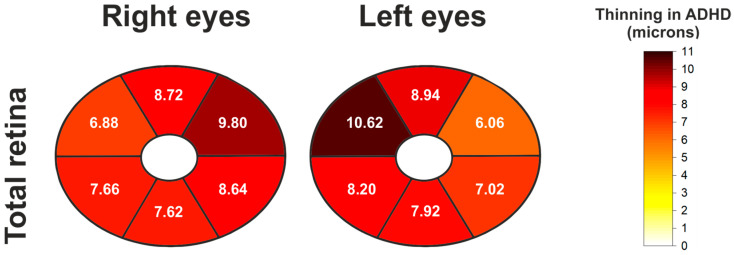
Schematic heatmaps of the macular elliptical grid showing statistically significant thinning (expressed in microns) of the total retina in the ADHD group compared to the control group, for both both right and left eyes. Mean thickness differences between groups (control group mean thickness minus ADHD group mean thickness) are indicated for each sector where significant differences were found. White sectors indicate no significant differences.

We next evaluated the total thickness, combining the inner (GCIPL + mRNFL) and outer retinal layer thicknesses. This analysis, also based on the elliptical segmentation pattern, reveals a significant and widespread reduction in ADHD patients across all sectors in both eyes. All comparisons reached statistical significance (*p* < 0.05), indicating a generalized thinning of the entire retinal profile in ADHD within the elliptical pattern (Table 6 and Figure 6).

### 3.8. AUC for Inner Retina and Outer Retina in Elliptical Pattern

AUC values were calculated to assess the diagnostic performance of total thickness (inner + outer layers) across all elliptical sectors in both eyes (Table 7). Most AUCs ranged between 0.6 and 0.7, suggesting low-to-moderate discriminative power. However, global values in both eyes, the inferior sector of the right eye, and the superonasal sector of the left eye achieved moderate diagnostic performance (AUC ≥ 0.7).

### 3.9. Comparisons of Peripapillary Retinal Nerve Fiber Layer (pRNFL) Thickness

No differences were detected between the two groups in the comparisons of the pRNFL, either in the quadrants or in the clock-hour sectors considered in the analysis.

### 3.10. Other Structural Parameters of the Optic Nerve

No statistically significant differences were observed between groups for rim area, disc area, average cup-to-disc ratio, vertical cup-to-disc ratio, cup volume, and disc diameter, indicating that these global structural parameters of the optic nerve were comparable in both populations.

## 4. Discussion

In this study, we investigated structural retinal changes in patients with ADHD compared to healthy controls using OCT, a non-invasive imaging modality that provides high-resolution cross-sectional views of retinal architecture. Our primary aim was to identify potential retinal biomarkers that may support early diagnosis and monitoring of ADHD, a neurodevelopmental condition affecting approximately 5–7% of the pediatric population and associated with considerable personal and societal burden [1,2].

ADHD has been widely studied through neuroimaging; however, OCT offers a cost-effective, rapid, and non-invasive window into the central nervous system via the retina, which originates embryologically from the neural tube. These tools are particularly well suited for populations with attentional difficulties, as they require minimal patient cooperation compared to modalities such as magnetic resonance imaging (MRI) [4].

### 4.1. Comparison with the Previous Literature

Our macular OCT findings reveal a generalized thinning of the total retinal thickness across all sectors in both eyes of ADHD patients. This supports the hypothesis of a bilateral, widespread retinal alteration in ADHD, aligning with reports by Sánchez et al. [5], who observed reduced macular thickness in ADHD patients.

The most notable thinning occurred in the outer retinal layers. These findings suggest a selective involvement of the outer retina, which may reflect altered neurodevelopment.

In contrast, inner retinal layers showed no changes, reinforcing the contribution of outer retinal thinning to the total retinal thinning.

Our findings are partially in line with previous studies. Erdogan et al. [6] and Ulucan et al. [7] reported reduced GCIPL and RNFL thicknesses in ADHD, possibly reflecting delayed cortical maturation. Conversely, Sujin et al. [8] reported increased macular thickness correlated with cortical changes, suggesting heterogeneity across populations and methodologies. Additionally, studies such as that by Tünel et al. [9] propose that structural retinal changes may become more pronounced with age, indicating progressive or persistent neurodevelopmental disruption.

At the optic nerve head, we found no significant changes in the peripapillary RNFL in ADHD patients. Two recent meta-analyses provide further context. Tonkaz et al. [10] reported global RNFL thinning in children with ADHD but no differences in ganglion cell layer or individual quadrants. In contrast, Bellato et al. [11] found no significant differences across retinal parameters. These discrepancies likely reflect methodological heterogeneity and underscore the need for standardized imaging protocols and larger sample sizes. Additionally, Kaymak et al. [12] found no correlation between RNFL thickness and ADHD symptom severity, suggesting retinal changes may not reflect clinical burden directly.

### 4.2. Clinical Implications

The potential of OCT as diagnostic tool in ADHD is promising but currently limited by modest discriminative performance. Receiver operating characteristic (ROC) analysis shown in the present study demonstrates an AUC between 0.6 and 0.7 for total retinal thickness, indicating low-to-moderate diagnostic accuracy. However, outer retinal thickness and combined measurements demonstrate higher discriminative ability in some sectors (AUC ≥ 0.7), particularly in the outer retina.

Although these findings are not sufficient for standalone diagnostic use, they may be valuable as part of a multi-modal assessment battery including clinical, neuropsychological, and imaging data—especially for screening purposes or monitoring therapeutic response.

### 4.3. Pathophysiological Insights

A selective reduction in the outer retinal layers and total macular thickness in ADHD, while the inner retina, GCIPL, mRNFL, pRNFL, and optic nerve parameters remain unchanged, suggests a localized alteration affecting primarily the photoreceptors, and/or bipolar cells. This finding is new, as most OCT studies in ADHD have focused on inner retinal layers, with mixed results.

Given the neurodevelopmental nature of ADHD, a potential explanation is that these changes could reflect subtle alterations in retinal neurodevelopment of the outer retina while not affecting the inner retina. In fact, the inner and outer retinal layers develop asynchronously over time [16].

Another possible factor is oxidative stress and low-grade neuroinflammation, which have been implicated in ADHD pathophysiology [17]. Photoreceptors are metabolically demanding and particularly susceptible to oxidative damage due to high oxygen consumption and exposure to light. A chronic imbalance between oxidative stress and antioxidant defenses in ADHD could selectively affect these outer retinal layers, leading to structural thinning, while sparing the inner retinal layers and optic nerve.

Additionally, subtle alterations in choroidal perfusion—the main blood supply for the outer retina—might play a role.

Nevertheless, these hypotheses are speculative and require further validation.

### 4.4. Limitations

Our study has several limitations. The small sample size and cross-sectional design restrict causal inference and generalizability. Imaging ADHD patients posed practical challenges due to cooperation issues, potentially affecting image quality. Technical limitations of OCT—such as motion artifacts and segmentation errors—also constrain data reliability. We did not evaluate inter-rater reliability among the specialists diagnosing ADHD in our sample. Although all cases met DSM-5 criteria confirmed by specialists, the lack of formal concordance data is a limitation and could be explored in future studies. Additionally, all participants were Caucasian and under the age of 26, limiting applicability to broader populations. Moreover, due to the automatic segmentation of the OCT device used, the outer retina defined in this study includes the region from the inner nuclear layer to Bruch’s membrane, making it impossible to determine which specific layer or layers are most directly affected by the thinning. Therefore, further studies with more refined segmentation capabilities are required. Another limitation is the reproducibility of OCT measurements. Test–retest variability of total retinal thickness has been reported to range between 2.25 and 5.88 microns across macular quadrants in the ETDRS grid when assessed by Cirrus OCT [18]. Since our total macular differences (4.50 to 10.40 microns) are slightly above this range, our results should be interpreted with caution. Finally, the influence of psychostimulant medication on retinal structure also warrants further investigation.

### 4.5. Strengths

Unlike previous research, we examined each eye separately, revealing consistent thinning in both the total and outer retina. The involvement of the outer retina emerges as a particularly relevant finding. The bilateral consistency of these results strengthens the potential of OCT as a tool for identifying potential structural markers in ADHD.

### 4.6. Future Directions

Future studies should aim to include larger, more diverse samples and adopt longitudinal designs to track retinal changes over time. The influence of psychostimulant and non-psychostimulant medication and its dosage on retinal structure also warrants exploration. Plus, the integration of machine learning tools for image analysis may enhance diagnostic performance and reveal novel patterns. Finally, future studies should investigate whether the degree of ADHD severity (mild, moderate, severe) and medication use are associated with the thinning observed. Such analyses were not conducted in the present study due to limitations in the available database.

## 5. Conclusions

This study demonstrates a diffuse reduction in macular thickness in individuals with ADHD compared to neurotypical controls, predominantly affecting the outer retinal layers. This structural thinning was consistently observed in both eyes. Moderate diagnostic performances in distinguishing ADHD from control subjects, as indicated by AUC values close to or exceeding 0.7, were obtained for these parameters. In contrast, the inner retinal layers, pRNFL, and optic nerve parameters did not show substantial differences.

These results suggest that structural OCT parameters may reflect subtle neurodevelopmental alterations in ADHD and could serve as complementary biomarkers in clinical and research settings. However, further longitudinal and multicenter studies with larger and more diverse populations are needed to validate their diagnostic utility and clinical relevance.

## Figures and Tables

**Table 1:** Comparisons of ophthalmic data between groups for the right and the left eyes. Best-corrected visual acuity (BCVA), spherical equivalent (SE), cycloplegia (cyclo), difference (dif.), attention deficit hyperactivity disorder (ADHD).

Right Eyes	Control Group	ADHD Group	Mean Dif.	*p* (T-Test)
**BCVA**	0.98 ± 0.06	0.95 ± 0.13	0.04	0.533
**SE pre cyclo**	−1.47 ± 2.52	−1.47 ± 2.78	0.01	0.996
**SE post cyclo**	−1.20 ± 2.56	−0.81 ± 2.51	0.38	0.445
**Axial length**	24.24 ± 1.05	24.12 ± 1.67	0.71	0.104
**K1**	42.08 ± 4.66	42.44 ± 1.57	−0.36	0.602
**K2**	43.68 ± 1.31	43.52 ± 1.71	0.59	0.163
**Left eyes**	**Control group**	**ADHD group**	**Mean dif.**	***p* (T-Test)**
**BCVA**	0.92 ± 0.08	0.96 ± 0.10	0.01	0.533
**SE pre cyclo**	−1.73 ± 2.46	−1.42 ± 2.97	0.30	0.579
**SE post cyclo**	−1.03 ± 2.47	−0.96 ± 3.05	4.07	0.251
**Axial length**	24.24 ± 1.02	24.12 ± 1.73	0.11	0.683
**K1**	42.65 ± 1.28	42.27 ± 1.75	0.37	0.227
**K2**	43.78 ± 1.40	43.54 ± 1.81	0.23	0.468

**Table 2:** ANCOVA adjusted for age and sex with Benjamini–Hochberg correction (BH) for multiple comparisons applied to total retinal thickness in ETDRS grid. Values are expressed as mean (microns) ± standard deviation (SD). Abbreviations: SD, standard deviation; BH, Benjamini–Hochberg correction; Diff., difference. All significant results are highlighted in bold.

Total Retinal Thickness (ETDRS Pattern)						
Eye	Location	Control Mean ± SD	ADHD Mean ± SD	Mean Diff. (Control-ADHD)	*p*-Value ANCOVA	*p*-Value ANCOVA + BH
Right	C0	265.96 ± 22.55	259.38 ± 22.04	6.58	0.1380	0.1380
	N1	332.70 ± 14.60	322.46 ± 16.00	10.24	0.0006	**0.0025**
	S1	329.34 ± 13.67	321.02 ± 14.16	8.32	0.0010	**0.0028**
	T1	316.38 ± 14.01	308.68 ± 14.89	7.7	0.0034	**0.0061**
	I1	326.74 ± 13.43	318.16 ± 15.86	8.58	0.0012	**0.0028**
	N2	302.46 ± 12.22	292.06 ± 20.77	10.4	0.0040	**0.0061**
	S2	282.16 ± 12.91	276.70 ± 13.03	5.46	0.0188	**0.0212**
	T2	264.38 ± 12.66	259.18 ± 14.22	5.2	0.0117	**0.0150**
	I2	271.24 ± 8.92	263.92 ± 14.13	7.32	0.0004	**0.0025**
Left	C0	265.18 ± 22.63	260.40 ± 22.13	4.78	0.2322	0.2322
	T1	315.14 ± 13.63	309.26 ± 15.05	5.88	0.0168	**0.0251**
	S1	330.30 ± 13.50	322.44 ± 14.55	7.86	0.0020	**0.0101**
	N1	332.96 ± 14.95	323.98 ± 16.42	8.98	0.0022	**0.0101**
	I1	325.18 ± 12.75	318.14 ± 16.43	7.04	0.0046	**0.0104**
	T2	263.70 ± 11.26	258.52 ± 14.96	5.18	0.0395	**0.0444**
	S2	282.62 ± 10.40	276.48 ± 13.80	6.14	0.0042	**0.0104**
	N2	302.44 ± 11.75	295.48 ± 16.41	6.96	0.0085	**0.0152**
	I2	269.70 ± 9.84	265.20 ± 14.72	4.5	0.0212	**0.0273**

**Table 3:** Area under the curve (AUC) for total retina thickness in ETDRS pattern for right and left eyes.

Total Retina (ETDRS Pattern)		
Sector	AUC Right Eyes	AUC Left Eyes
C0	0.58	0.58
N1	0.68	0.68
N2	0.66	0.66
S1	0.66	0.66
S2	0.61	0.61
T1	0.65	0.65
T2	0.61	0.61
I1	0.67	0.67
I2	0.69	0.69

**Table 4:** ANCOVA adjusted for age and sex with Benjamini–Hochberg correction (BH) for multiple comparisons applied to outer retinal thickness in the elliptical grid. Values are expressed as mean (microns) ± standard deviation (SD). Abbreviations: SD, standard deviation; BH, Benjamini–Hochberg correction; Diff., difference. All significant results are highlighted in bold.

Outer Retinal Thickness (Elliptical Pattern)						
Eye	Location	Control Mean ± SD	ADHD Mean ± SD	Mean Diff. (Control-ADHD)	*p*-Value ANCOVA	*p*-Value ANCOVA + BH
Right	Global	128.74 ± 6.60	121.98 ± 8.67	6.76	<0.0001	**<0.0001**
	Superotemporal	129.14 ± 7.33	123.14 ± 8.85	6.00	0.0003	**0.0004**
	Superior	133.46 ± 7.84	125.98 ± 9.79	7.48	0.0001	**0.0001**
	Superonasal	130.72 ± 9.36	122.38 ± 10.80	8.34	0.0002	**0.0002**
	Inferonasal	127.30 ± 8.63	119.94 ± 8.89	7.36	<0.0001	**<0.0001**
	Inferior	125.10 ± 6.90	119.18 ± 9.07	5.92	<0.0001	**<0.0001**
	Inferotemporal	127.10 ± 7.26	121.20 ± 9.44	5.90	0.0001	**0.0002**
Left	Global	128.42 ± 6.37	122.98 ± 8.74	5.44	0.0002	**0.0012**
	Superotemporal	129.24 ± 6.70	125.16 ± 9.93	4.08	0.0082	**0.0082**
	Superior	133.28 ± 7.81	126.70 ± 10.30	6.58	0.0005	**0.0012**
	Superonasal	130.50 ± 10.43	122.70 ± 10.62	7.80	0.0005	**0.0012**
	Inferonasal	126.16 ± 8.49	120.98 ± 8.72	5.18	0.0011	**0.0018**
	Inferior	124.50 ± 6.00	120.04 ± 9.49	4.46	0.0006	**0.0012**
	Inferotemporal	126.80 ± 6.09	122.54 ± 9.63	4.26	0.0046	**0.0053**

**Table 5:** Area under the curve (AUC) for outer retinal thickness in elliptical pattern for right and left eyes.

Outer Retina (Elliptical Pattern).		
Sector	AUC Right Eyes	AUC Left Eyes
Global	0.73	0.71
Superior	0.72	0.70
Superotemporal	0.69	0.62
Inferotemporal	0.69	0.65
Inferior	0.70	0.68
Inferonasal	0.73	0.66
Superonasal	0.73	0.72

**Table 6:** ANCOVA adjusted for age and sex with Benjamini–Hochberg correction (BH) for multiple comparisons applied to total retina thickness (inner retina + outer retina) in elliptical grid. Values are expressed as mean (microns) ± standard deviation (SD). Abbreviations: SD, standard deviation; BH, Benjamini–Hochberg correction; Diff., difference. All significant results are highlighted in bold.

Total Retinal Thickness (Elliptical Pattern)						
Eye	Location	Control Mean ± SD	ADHD Mean ± SD	Mean Diff. (Control-ADHD)	*p*-Value ANCOVA	*p*-Value ANCOVA + BH
Right	Global	244.76 ± 10.54	236.62 ± 12.17	8.14	0.0005	**0.0020**
	Superotemporal	233.0 ± 9.75	226.12 ± 11.65	6.88	0.0028	**0.0031**
	Superior	251.98 ± 10.79	243.26 ± 13.1	8.72	0.0016	**0.0025**
	Superonasal	253.74 ± 11.55	243.94 ± 13.47	9.8	0.0005	**0.0020**
	Inferonasal	251.1 ± 12.72	242.46 ± 12.91	8.64	0.0008	**0.0020**
	Inferior	243.96 ± 15.86	236.34 ± 13.66	7.62	0.0016	**0.0025**
	Inferotemporal	234.7 ± 11.01	227.04 ± 14.69	7.66	0.0025	**0.0031**
Left	Global	245.4 ± 8.64	237.18 ± 12.94	8.22	0.0002	**0.0008**
	Superotemporal	234.74 ± 9.14	228.68 ± 13.17	6.06	0.0036	**0.0041**
	Superior	252.64 ± 9.81	243.7 ± 14.58	8.94	0.0005	**0.0008**
	Superonasal	253.86 ± 12.1	243.24 ± 14.65	10.62	0.0003	**0.0008**
	Inferonasal	250.86 ± 10.57	242.66 ± 13.22	8.2	0.0006	**0.0009**
	Inferior	244.18 ± 9.47	236.26 ± 14.69	7.92	0.0003	**0.0008**
	Inferotemporal	235.16 ± 8.7	228.14 ± 15.01	7.02	0.0043	**0.0043**

**Table 7:** Area under the curve (AUC) for outer retina and inner retina thickness (total retina thickness) in elliptical pattern for right and left eyes.

Total Retina (Elliptical Pattern)		
Sector	AUC Right Eyes	AUC Left Eyes
Global	0.70	0.70
Superior	0.69	0.69
Superotemporal	0.67	0.65
Inferotemporal	0.66	0.65
Inferior	0.70	0.68
Inferonasal	0.69	0.68
Superonasal	0.69	0.72

## Data Availability

The data sets generated and/or analyzed during this study are available from the corresponding author on reasonable request.
